# Efficacy of a melatonin receptor agonist and orexin receptor antagonists in preventing delirium symptoms in the olderly patients with stroke: a retrospective study

**DOI:** 10.1186/s40780-024-00397-z

**Published:** 2024-11-18

**Authors:** Yukiko Miyoshi, Yuki Shigetsura, Daiki Hira, Takakuni Maki, Hirotsugu Kawashima, Naoko Sugita, Noriko Sugawara, Noriaki Kitada, Machiko Hirai, Masayoshi Kawata, Hiroki Endo, Yusuke Kojima, Keiko Ikuta, Yurie Katsube, Natsuki Imayoshi, Shunsaku Nakagawa, Masahiro Tsuda, Tomohiro Terada

**Affiliations:** 1 Department of Clinical Pharmacology and Therapeutics, Kyoto University Hospital, 54 Shogoin- Kawahara-cho, Sakyo-ku, Kyoto, 606-8507 Japan; 2 Department of Neurology, Graduate School of Medicine, Kyoto University, 54 Shogoin- Kawahara-Cho, Sakyo-Ku, Kyoto, 606-8507 Japan; 3 Department of Psychiatry, Graduate School of Medicine, Kyoto University, 54 Shogoin- Kawahara-Cho, Sakyo-Ku, Kyoto, 606-8507 Japan; 4 Department of Nursing, Kyoto University Hospital, 54 Shogoin-Kawahara-Cho, Sakyo-Ku, Kyoto, 606-8507 Japan; 5 Graduate School of Pharmaceutical Sciences, Kyoto University, 46-29 Yoshida-Shimo- Adachi-cho, Sakyo-Ku, Kyoto, 606-8501 Japan

**Keywords:** Stroke, Delirium, Melatonin receptor agonist, Orexin receptor antagonist

## Abstract

**Background:**

Post-stroke delirium affects between 24% and 43% of patients, and negatively impacts patient outcomes. Recently, research attention has been on preventive interventions for delirium, with melatonin receptor agonists and orexin receptor antagonists reported to be effective in preventing delirium in intensive care unit patients. However, the efficacy of these agents in preventing post-stroke delirium remain unclear. This study examined the efficacy of ramelteon, suvorexant, and lemborexant in preventing post-stroke delirium symptoms in patients with stroke.

**Methods:**

A retrospective survey of medical records was conducted for patients with stroke aged > 75 years at Kyoto University Hospital from October 2021 to March 2023. Patients who received ramelteon, suvorexant, or lemborexant on admission and the following day were classified into the consecutive administration group, whereas those who did not were classified into the non-consecutive administration group. The primary outcome was an increase in the number of positive items in the delirium screening tool over 7 days.

**Results:**

Of the 104 patients, 33 and 71 were in the consecutive and non-consecutive administration groups, respectively. Fewer patients in the consecutive administration group had an increase in the number of positive items than in the other group (6% vs. 21%). Patients in the consecutive administration group significantly less often had an increase in the number of positive items in the delirium screening tool (*P* = 0.05; hazard ratio, 0.27; 95% confidence interval, 0.10–0.75).

**Conclusions:**

This study revealed that early administration of a melatonin receptor agonist or orexin receptor antagonists may effectively prevent post-stroke delirium in older patients.

**Supplementary Information:**

The online version contains supplementary material available at 10.1186/s40780-024-00397-z.

## Background

Stroke is the second leading cause of death worldwides, as well as the third leading cause of death and disability combined [1]. Furthermore, this condition is one of the major causes of death in older adult patients, with nearly one-third of all strokes occurring in patients aged 75 years and older [2, 3], and more than half of those patients experiencing reduced mobility [3]. Therefore, with an aging population, the treatment of stroke in older individuals is increasingly vital. In the pharmacological treatment of stroke, initiating antiplatelet therapy with aspirin within 48 h of onset is effective in improving long-term outcomes [4–6]. Furthermore, initiating rehabilitation as early as possible, particularly intensive rehabilitation within 24 h of onset, significantly improves functional disabilities [7]. Consequently, prompt initiating of both pharmacological treatment and rehabilitation following stroke onset is essential for improving outcomes.

Stroke is a common cause of delirium [8]. The incidence of delirium in the acute phase following stroke ranges from 24 to 43% of cases [9–14], with a meta-analysis utilizing a random-effects approach placing the rate at 26% (95% confidence interval [CI]: 19–33%) [9]. Delirium is an independent risk factor for death and the later onset of dementia [15]. It also presents a patient safety concern, as it can lead to dangerous behaviors and falls, while also interfering with adherence to treatment of the underlying disease [16] and rehabilitation. These may hamper early initiation of pharmacotherapy and rehabilitation after stroke onset, resulting in worsening outcomes. Therefore, it is crucial to implement appropriate prophylaxis and interventions for delirium promptly.

In a randomized controlled trial and a retrospective observational study for patients in intensive care units, ramelteon (a melatonin receptor agonist) [17], suvorexant [18], and lemborexant [19] (orexin receptor antagonists) administered nightly to older patients admitted for acute care were reported to prevent the onset of delirium in patients. However, these reports included various diseases, such as infection, heart failure, and stroke, and the effectiveness of these sleep medications in patients with stroke has not been verified. In daily practice, these medications are commonly utilized for sleep management following the onset of stroke because approximately 20 to 40% of patients with stroke have sleep-wake disorders [20]. We determined that it is possible to investigate the effects of ramelteon, suvorexant, and lemborexant on post-stroke delirium in older patients based on the medical records. In this study, we retrospectively investigated the effects of these three kinds of sleep medications on prevention of delirium symptoms.

## Methods

### Patients

Patients with stroke, aged 75 years or older, admitted for acute care to the Department of Neurology, Kyoto University Hospital between October 2021 and March 2023 were retrospectively analyzed. The patients followed up to one week after admission. Patients who were transferred to another hospital, died within a week of admission, or could not be evaluated due to communication difficulties arising from stroke-related symptoms, such as impaired consciousness, were excluded.

Patients were categorized into two groups: a consecutive administration group and a non-consecutive administration group. The consecutive administration group consisted of patients who received ramelteon, suvorexant, and lemborexant on the day of admission and the following before sleep, because of complaints of insomnia. In contrast, the non-consecutive administration group consisted of patients who were not treated with a melatonin receptor agonist or orexin receptor antagonist on admission or the following day. Previous studies have highlighted the importance of initiating treatment within 48 h of stroke onset [4–6], making it essential to investigate the effect of early intervention on preventing delirium. Additionally, prolonged use of hypnotics is associated with an increased risk of adverse events, such as falls, suggesting the importance of evaluating their short-term efficacy. Therefore, in this study, patients were divided into these two groups based on a 2-day drug administration period. The consecutive administration group also included patients who continued using hypnotics for more than 3 days depanding on their sleep status.

### Assessment of delirium

Delirium was assessed by doctors or nurses using the Delirium Screening Tool (DST) [21, 22], which is based on the Delirium Rating Scale (DRS) [23]. The DST has a sensitivity of 98% and has been verified as a useful screening tool. The DST comprises 11 items, belonging to the following categories: (A) level of consciousness, arousal, and environmental awareness (seven items), (B) change in cognition (two items), and (C) change in symptoms (two items). If patients were positive for any item in category A, then patients were evaluated for category B items. If patients were positive for any item in category B, then they were evaluated for category C items. In this study, we investigated an increase in the number of items from category A, which all patients were evaluated for, including a sense of reality, decrease in activity, excitement, mood fluctuation, sleep–awakening level, delusion and hallucination. Given that DST evaluations are conducted according to standardized manuals, the likelihood of variability in results among evaluators is minimized. It has been reported that more than 90% of patients who develop delirium after a stroke do so within 4 days of onset [12]. To cover this period, a 7-day observation period was set in this study.

### Variables and measurement

In addition to the DST, information on age, sex, cognitive impairment, and other delirium risk factors, history of delirium, medications associated with an increased risk of delirium, and regular alcohol use were extracted from the medical records. The medications were only listed if they were administered to two or more patients. Adverse events, including self-extraction of the intravascular tube, falls, somnolence, drowsiness, lightheadness, dizziness, hepatic impairment, and renal impairment attributed to sleep medications and delirium, were investigated during the first seven days following admission. In this study, hepatic impairment was defined as ≥ 5 × upper limit of normal (ULN) elevation in alanine transaminase (ALT), ≥ 2 × ULN elevation in alkaline phosphatase (ALP) or ≥ 3 × ULN elevation in ALT and simultaneous elevation of total bilirubin (TBL) concentration exceeding 2 × ULN [24], and renal impairment was defined as serum creatinine levels that were 1.5 × the basesline, an increase of ≥ 0.3 mg/dL or urine volume ≤ 0.5 ml/kg/h for 6 h [25].

### Statistical analysis

Kaplan-Meier curves were used to estimate the time to increase in the number of positive items in category A of DST over 7 days. The log rank test (Mantel-Cox) as well as hazard ratio with 95% confidence intervals (CIs) were used to compare the two survival curves. Classified data from two independent populations were compared using the Fisher’s exact test. All data were analyzed using GraphPad Prism (version 9.5.0, GraphPad Software, San Diego, CA, USA). Statistical significance was set at a two-sided P value of < 0.05.

## Results

### Baseline characteristics

A total of 104 patients were eligible for this study; 33 patients were categorized into the consecutive administration group and 71 into the non-consecutive administration group. Table 1 shows the patients’ characteristics information at admission. Patients in the consecutive administration group were significantly more likely to have a history of delirium and cognitive decline than those in the non-consecutive administration group, both of which have been identified as risk factors for the development of delirium. No significant differences were observed in other risk factors between the groups. In the consecutive administration group, 20 patients were treated with a melatonin receptor agonist, 3 were treated with an orexin receptor antagonist, and 10 with a combination of both. Only 4 patients had been using these sleep medications prior to admission, while most patients commenced treatment after admission. Benzodiazepine receptor agonist treatment was continued in two patients, while its use was discontinued in three patients on admission. Of the two patients who continued treatment, both ramelteon and lemborexant were additionally prescribed in one patient, while the other was prescribed suvorexant. In the non-consecutive administration group, 7 out of 14 patients discontinued benzodiazepine receptor agonists, while 8 patients continued treatment at the same dose. While benzodiazepines are well-known risk factors for delirium, there was no difference in the frequency of their use between the two groups in this study. Admission blood laboratory values are presented in Table S1; however, complete data could not be obtained for all eligible patients.

**Table 1:** Baseline characteristics

Characteristics	Consecutive administration group	Non-consecutive administration group	*p* value
All patients, n	33	71	
Age, median (min-max), year	84 (75–96)	81 (75–94)	
Male, n (%)	19 (57.6)	39 (54.9)	0.83
Risk factor of delirium, n (%)			
Dementia	6 (18.2)	7 (9.8)	0.34
Cognitive decline	17 (51.5)	21 (29.6)	0.048
History of delirium	6 (18.2)	3 (4.2)	0.027
Habitual use of alcohol	3 (9.1)	9 (12.7)	0.74
Prescribed medication			
Benzodiazepine receptor agonist	5 (15.2)	14 (19.7)	0.79
Opioid	1 (3.0)	1 (1.4)	0.54
Corticosteroid	2 (6.1)	4 (5.6)	> 0.99
Histamine-2 receptor antagonist	2 (6.1)	3 (4.2)	0.65
Antiseizure medication	1 (3.0)	1 (1.4)	0.54
Antiparkinsonism agent	2 (6.1)	1 (1.4)	0.24
Hypnotics added upon admission, n			
Ramelteon 8 mg	30	-	-
Lemborexant 2.5 mg 5 mg	3 8	-	-
Suvorexant 10 mg 15 mg	1 1	-	-
DST positive items at day 0, n			
Abnormal sense of reality	1	0	0.31
Decreased activity	0	1	> 0.99
Excitement	3	1	0.093
Mood fluctuations	1	0	0.31
Sleep-wake rhythms	4	4	0.26
Delusions	1	0	0.31
Hallucinations	0	0	-

Baseline parameters were collected on admission. P values reflect the difference between the consecutive administration group and the non-consecutive administration group at beseline using the Fisher’s exact test

### Delirium assessment

**Fig. 1:**
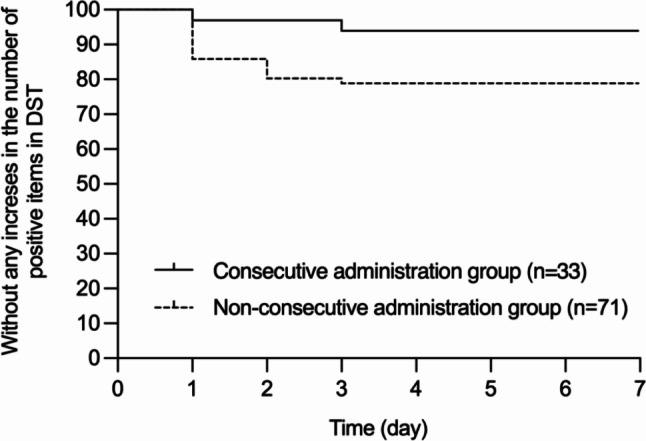
Time to increase in the number of positive items of category A in the Delirium Screening Tool. Kaplan-Meier curves were used to estimate the time to increase in the number of positive items in category A of the Delirium Screening Tool (DST) over 7 days. The log rank test (Mantel-Cox) as well as hazard ratio with 95% confidence intervals (CIs) were used to compare the two survival curves. (*P* = 0.05; hazard ratio, 0.27; 95% confidence interval, 0.10–0.75)

**Fig. 2:**
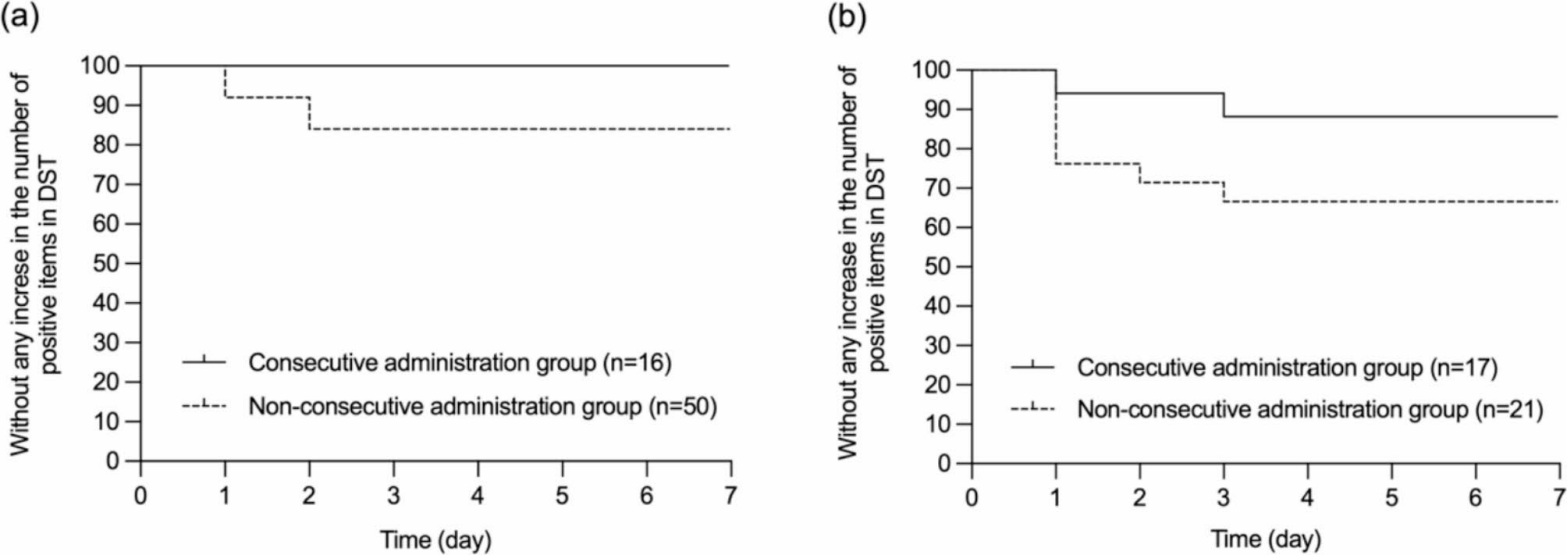
Time to increase in the number of positive items in category A in the Delirium Screenig Tool in the patients without or with Cognitive Decline. Kaplan-Meier curves were used to estimate the time to increase in the number of positive items in category A of the Deliruim Screening Tool (DST) in the patients without (a) or with (b) cognitive decline over 7 days. The log rank test (Mantel-Cox) as well as hazard ratio with 95% confidence intervals (CIs) were used to compare the two survival curves. (Fig. 2a: *P* = 0.11, 95% confidence interval, 0.09–1.20; Fig. 2b: *P* = 0.09)

Two patients (6.1%) in the consecutive administration group and 15 patients (21.1%) in the non-consecutive administration group had an increase in the number of positive items from category A of the DST compared to their status at admission. Of the two patients in the consecutive administration group, one was treated with suvorexant, while the other was treated with a combination of ramelteon and lemborexant. Figure 1 shows the Kaplan-Meier curves for the time to increase in the number of positive items in category A of DST over 7 days. The log-rank test (Mantel-Cox) showed that significantly less in patients in the consecutive administration group had an increase in the number of positive items in the category A of DST than in those in the non-consecutive administration group (*P* = 0.05, hazard ratio: 0.27, 95% CI: 0.10–0.75). The same analysis was performed for the two groups with or without cognitive decline in cognitive function at the time of admission (Fig. 2). Irrespective of cognitive decline status, fewer patients in the consecutive administration group had an increase in the number of positive items in the category A of DST than those in the non-consecutive administration group (Fig. 2a: *P* = 0.11, 95% CI: 0.09–1.20, Fig. 2b: *P* = 0.09).

### Sleep-awakening level from the DST

Since this study focused on sleep medications, the “Sleep-awakening level” item, depending on the presence or absence of a sleep disorder, from the DST was analyzed. In the consecutive administration group, four patients (12.1%) were positive for the “Sleep-awakening level” item on day 0, and one patient (3.0%) was positive on day 3. Conversely, in the non-consecutive administration group, four patients (5.6%) were positive on day 0 and nine patients (12.7%) were positive on day 3. Furthermore, two patients in the consecutive administration group and 12 in the non-consecutive administration group exhibited an increase in items other than the “sleep-awakening level” (Table S2).

### Safety

The adverse events associated with sleep medication use and delirium are presented in Table 2. No significant differences were observed in these adverse events between the two groups. However, self-extraction of intravascular tube resulting from delirium occurred more frequently in the consecutive administration group (15.2%) than in the non-consecutive administration group (7.0%).

**Table 2:** Comparison of adverse events associated with sleep medication use and delirium

Adverse events, *n* (%)	Consecutive administration group *n* = 33	Non-consecutive administration group *n* = 71	*p* value
Somnolence	6 (18.2)	11 (15.5)	0.78
Drowsiness	1 (3.0)	2 (2.8)	> 0.99
Lightheadedness	8 (24.2)	15 (21.1)	0.80
Dizziness	0 (0)	4 (5.6)	0.30
Hepatic impairment	0 (0)	1 (1.4)	> 0.99
Renal impairment	2 (6.1)	2 (2.8)	0.58
Self-extraction of intravascular tube	5 (15.2)	5 (7.0)	0.28

## Discussion

This study suggests that the use of a melatonin receptor agonist or orexin receptor antagonists during the first 2 days after admission could potentially prevent symptoms of delirium. It is noteworthy that patients in the consecutive administration group who had more risk factors for delirium, including cognitive decline and history of delirium [8, 26, 27], exhibited less symptoms of delirium. It is also possible that cognitive decline and a history of delirium influenced the decision to prescribe sleep medications, as there is a strong association between cognitive decline and sleep disturbances in older adults [28]. In this study, although these medication were administered for insomnia, they may indirectly contributed to the prevention of delirium. These findings align with those observed in patients from previously reported studies [17, 18, 29, 30], suggesting that the use of melatonin receptor agonist or orexin receptor antagonists could potentially lower the risk of delirium.

Notably, the number of patients who tested positive for the DST “Sleep-Awakening Level” item decreased in the consecutive administration group but increased in the non-consecutive administration group. Due to the limited sample size of this study, a comprehensive analysis of the relationship between Sleep-Awakening Level item disturbances and delirium was not feasible. However, sleep medication appeared to improve insomnia, a known predisposing factor for delirium [26]. Furthermore, the positive results for items other than “Sleep-Awakening Level” in the non-consecutive administration group suggested that the results are related to delirium as well as improved sleep control. Thus, it potentially decrease the risk of delirium. This preliminary observation highlights the need for further research on the effect of sleep management on the incidence of delirium in patients with stroke.

This retrospective study revealed that 21% of patients in the non-consecutive administration group who did not receive the melatonin receptor agonist or orexin receptor antagonist for two days had an increase in the number of positive items of DST. This incidence aligns closely with that reported in a previous meta-analysis (26%) [9], though a direct comparison with previous studies is challenging due to differences in assessment methods. Additionally, the timing of increase in the number of positive items observed in this study mirrors that reported in prior research [12], with all cases manifesting within four days. These results suggest that this DST-based assessment serves as a valid tool for detection of delirium, rendering the findings of this study as having significant importance.

Sleep-wake rhythm disturbances may play a role in the pathological mechanisms underlying delirium, and could also be a contributing factor [31]. Consequently, effective management of sleep through the administration of melatonin receptor agonists and orexin receptor antagonists is considered highly beneficial in the prevention of delirium. Furthermore, evidence indicates that inflammatory cytokines are also involved in the pathogenesis of delirium [32]. A study on patients with delirium found that interleukin-6 (IL-6) levels were significantly lower in patients treated with melatonin than in those receiving a placebo [33]. Additionally, increased serum orexin levels have been reported in patients with delirium [34]. These findings suggest that melatonin receptor agonists and orexin receptor antagonists may contribute to the prevention of delirium through improved sleep management and pharmacological effects. However, the present study did not assess inflammatory cytokines or other relevant serum factors, and further research is required to elucidate these underlying molecular mechanisms.

Considering the risk of adverse events associated with sleep medication [35], there were concerns regarding the older adult participants in this study. In particular, orexin receptor antagonists may increase the risk of falls [36] and should be prescribed with caution, especially in this population. However, no significant difference was observed in the frequency of adverse events between the consecutive and non-consecutive administration group. One potential explanation for this outcome is that the treatment protocol for cerebral infraction often includes behavioral restrictions following the initiation of treatment [37], which could have suppressed the occurrence of behavior-related adverse events, such as falls. This investigation focused on the short-term sleep medication (limited to two days post-admission). To our knowledge, only one small randomized control trial has been reported on strategies for the prevention of delirium in the acute phase of stroke [38]. Our findings suggest that the short-term use of a melatonin receptor antagonist and orexin receptor agonists in patients with stroke may be safe and could potentially contribute to preventing further episode of delirium. However, delirium is often associated with long-term consequences, such as an increased risk of dementia and mortality [15]. Therefore, further research is necessary to determine the long-term effects of a melatonin receptor agonist or orexin receptor antagonists in patients following a stroke.

The limitations of this study are as follows. First, this study was a single-center, retrospective study. Therefore, it was not possible to generalize the patient backgrounds, which resulted in differences in items such as cognitive decline and history of delirium. Conversely, the singular nature of the study site ensured uniformity in the hospitalization environment and a cadre of healthcare providers, potentially suppressing the variance in factors known to influence delirium outcomes. Second, the sample size in this study was relatively small. A post-hoc analysis of statistical power based on the study findings (hazard ratio = 0.27, total number of events over both groups = 17, alpha = 0.05, using the R package “powerSurvEpi”) showed a low statistical power of 0.44. The sample size required to guarantee a statistical power of 0.8 is approximately 250. Therefore, future studies should employ a larger sample size to more effectively assess the impact of the study drug. Due to the limited sample size, it was not feasible to analyze the data by grouping patients based on the specific drug administered. Given that melatonin receptor agonist and orexin receptor antagonists have distinct pharmacological mechanisms, their effects on delirium prevention may also differ. Consequently in line with previous studies, we analyzed a melatonin receptor agonist and orexin receptor antagonists as a single group in this study. Third, it should be noted that a widely accepted rating scale such as the DRS-R-98 [39] was not employed in assessing delirium. Therefore, the incidence and severity of delirium could not be assessed accurately. Nevertheless, as previously mentioned, the findings of this study are deemed significant because of their validity in comparison with past research findings [12, 17–19] regarding the efficacy of those sleep medication in preventing delirium and the timing of its onset. Additional research utilizing scales such as the DRS-R-98 [39] is required to further validate of these results.

## Conclusion

In this retrospective and single-center analysis, we demonstrated that administering a melatonin receptor agonist or orexin receptor antagonists during the first 2 days of hospital admission can effectively reduce the symptoms of delirium in older patients with stroke.

## Electronic supplementary material

Below is the link to the electronic supplementary material.

Supplementary Material 1

## Data Availability

Not applicable.

## Abbreviations

DST: Delirium Screening Tool
ULN: Upper limit of normal
ALT: Alanine transaminase
ALP: Alkaline phosphatase
TBL: Total bilirubin
