# Supplementary material for: Efficacy of a melatonin receptor agonist and orexin receptor antagonists in preventing delirium symptoms in the olderly patients with stroke: a retrospective study
Source: J Pharm Health Care Sci. 2024 Nov 18;10:74. doi: 10.1186/s40780-024-00397-z (PMC11572110; doi:10.1186/s40780-024-00397-z)
Supplement: Supplementary file 1 — Supplementary Material 1 [file 40780_2024_397_MOESM1_ESM.pdf]

Table S1. Laboratory values upon admission

| Laboratory values, Mean (SD) | Consecutive administration group n=33 | Non-consecutive administration group n=71 | *p* value |
|---|---|---|---|
| Na (mmol/L) | 139.4 (2.9) n=33 | 139.5 (3.4) n=71 | 0.82 |
| K (mmol/L) | 4.1 (0.6) n=33 | 4.0 (0.4) n=71 | 0.25 |
| Ca (mg/dL) | 8.9 (0.4) n=28 | 9 (0.4) n=60 | 0.24 |
| Mg (mg/dL) | 2.0 (0.3) n=10 | 2.0 (0.3) n=16 | 0.82 |
| CRP (mg/dL) | 0.6 (2.0) n=33 | 1.1 (4.1) n=71 | 0.49 |

Comparisons between the consecutive administration group and the non-consecutive administration group were conducted using the unpaired *t*-test.

Table S2. Patients with an increase in the number of positive DST items within 7 Days

| | Consecutive administration group | Non-consecutive administration group |
|---|---|---|
| Patients with an increase in the number of positive DST items, n | 2 | 15 |
| DST items, n | | |
| Abnormal sense of reality | 1 | 1 |
| Decreased activity | 1 | 3 |
| Excitement | 1 | 6 |
| Mood fluctuations | 1 | 6 |
| Sleep-wake rhythms | 1 | 8 |
| Delusions | 0 | 0 |
| Hallucinations | 1 | 0 |
